# Modeling the Contribution of Male Testosterone Levels to the Duration of Positive COVID Testing among Hospitalized Male COVID-19 Patients

**DOI:** 10.3390/diagnostics11040581

**Published:** 2021-03-24

**Authors:** Stefano Salciccia, Michael L. Eisenberg, Martina Maggi, Silvia Lai, Claudio Maria Mastroianni, Patrizia Pasculli, Maria Rosa Ciardi, Vittorio Canale, Matteo Ferro, Gian Maria Busetto, Ettore De Berardinis, Gian Piero Ricciuti, Alessandro Sciarra, Francesco Del Giudice

**Affiliations:** 1 Department of Maternal-Infant and Urological Sciences, “Sapienza” University of Rome, Policlinico Umberto I Hospital, 00161 Rome, Italy; stefano.salciccia@uniroma1.it (S.S.); martina.maggi@uniroma1.it (M.M.); vittorio.canale@uniroma1.it (V.C.); ettore.deberardinis@uniroma1.it (E.D.B.); gianpiero.ricciuti@uniroma1.it (G.P.R.); alessandro.sciarra@uniroma1.it (A.S.); francesco.delgiudice@uniroma1.it (F.D.G.); 2 Department of Urology, Stanford University School of Medicine, Stanford, CA 94305, USA; eisenberg@stanford.edu; 3 Department of Translational and Precision Medicine, “Sapienza” University of Rome, Policlinico Umberto I Hospital, 00161 Rome, Italy; silvia.lai@uniroma1.it; 4 Department of Public Health and Infectious Diseases, “Sapienza” University of Rome, Policlinico Umberto I Hospital, 00161 Rome, Italy; claudio.mastroianni@uniroma1.it (C.M.M.); patrizia.pasculli@uniroma1.it (P.P.); mariarosa.ciardi@uniroma1.it (M.R.C.); 5 Department of Urology, European Institute of Oncology (IEO), IRCCS, 20141 Milan, Italy; 6 Department of Urology and Organ Transplantation, University of Foggia, 71122 Foggia, Italy; gianmaria.busetto@unifg.it

**Keywords:** COVID-19, total testosterone, male serum testosterone, viral positivity duration, COVID-19 testing

## Abstract

**Background:** A growing body of evidence is emerging suggesting testosterone can affect all cells involved in the immune response to both bacterial and viral infections, and the testosterone effect on the immune response could explain the greater susceptibility of men to infections including COVID-19. We aimed to explore the predictive role of male serum total testosterone (TT) levels on the time till viral negativity testing among hospitalized COVID-19 patients. **Methods:** The univariate effect of risk factors for the duration of COVID-19 viral positivity was evaluated using the log-rank test and Kaplan–Meier estimates. A multivariable Cox regression model was developed to test the role of TT levels and the subsequent odds for shorter viral positivity intervals. **Results:** Increasing serum TT levels and the need for an oxygen administration strategy were independently predictive for respectively reduced and increased days to negativization (Hazard Ratio [HR]: 1.39, 95% CI: 0.95–2.03 and HR: 0.19, 95% CI: 0.03–1.18). **Conclusion:** Baseline higher TT levels for male COVID-19 patients at hospital admission are associated with shorter durations of positive COVID-19 testing and thus viral clearance. Our preliminary findings might play a relevant to help pandemic control strategies if these will be verified in future larger multicentric and possibly randomized trials.

## 1. Introduction

The pandemic caused by the respiratory syndrome coronavirus 2 (SARS-CoV-2), which leads to COVID-19, is devastating the health systems around the world with geographical variations [1]. Differences in the incidence and mortality could be explained by the spread of the virus, by the different measures of social distancing adopted by the various countries, and also by differences in populations’ susceptibility to infection [2]. Since the beginning of the pandemic, it has been shown that the clinical presentation of patients with COVID-19 infection varies from asymptomatic infection to death [3]. Several risk factors including age, comorbidities, and sex have been proposed to explain this variability of clinical presentation [4,5]. Studies indicate male sex and older age to be significant risk factors for severe COVID-19 infection regardless of the geographical area and suggest that the hormonal constitution could play a role both in the susceptibility and severity of the clinical course [6]. In this context, there is evidence that testosterone can affect all cells involved in the immune response to both bacterial and viral infections, and the testosterone effect on the immune response could explain the greater susceptibility of men to infections including COVID-19 [7,8,9]. Indeed, previous clinical experiences in patients with COVID-19 infection showed that testosterone levels were significantly lower in patients with a more severe clinical course [10,11] suggesting a role of testosterone in the immune response to COVID-19 [12,13,14]. While the risk factors for COVID-19 severity have been evaluated, less data on factors associated with viral clearance have been studied. Knowledge of viral positivity duration is a crucial factor for an epidemiological control strategy. Therefore, the aim of our study was to evaluate the association of male serum total testosterone (TT) levels and the duration of positive SARS-CoV-2 viral testing among hospitalized COVID-19 patients.

## 2. Materials and Methods

After Institutional Review Board (IRB) approval, we performed a retrospective analysis of our prospectively maintained database of patients who were hospitalized due to COVID-19 infection between March 2020 and June 2020. Patients were considered eligible if they had a positive SARS-CoV-2 test by real-time reverse transcriptase–polymerase chain reaction (RT-PCR) at the moment of hospital admission. Daily viral testing (sampling interval of ≥1 day) was performed with RT-PCR during the range of hospital stay. A return to negative results for SARS-CoV-2 testing (sampling interval of ≥1 day) was determined with at least two consecutive RT-PCR negative results. The COVID-19 positivity duration was defined as the timeframe between the first confirmatory result at admission and the day of the two consecutive RT-PCR negative tests during the range of hospitalization. Patient medical history and routine laboratory test data, including counts of peripheral white blood cells, immune cell subsets, cytokine interleukin (IL) 6, and TT levels were matched with the day of the primary confirmatory RT-PCR result. The severity of clinical manifestations was assessed at admission on the basis of oxygen requirements (i.e., Ventimask or CPAP) and clinical symptoms such as fever and respiratory tract pneumonia (identified on imaging).

### Statistical Analysis

Descriptive statistics were used to summarize pertinent study information. The association between variables was tested by the values according to Fisher’s Exact test or Mann–Whitney U test when appropriate. The differences in duration of viral positivity were initially explored using the Log-rank test and graphically presented as a Kaplan–Meier curves estimate. A univariate Cox proportional hazard model was developed by testing each potential risk factor (both dichotomized and continuous variable) as a covariate for time to RT-PCR negative result with significance set at *p* ≤ 0.05. A multivariable stepwise regression model (forward selection) was further generated, by selecting those predictive variables that were significant upon univariate analysis, with entering and removing limits set at *p* = 0.05 and *p* = 0.10, respectively. Given that impaired TT levels are a function of different confounders rather than a pre-established threshold, we also modeled the multivariate effect of serum TT according to the Age-Adjusted Charlson Comorbidity Index (ACCI), history of hypertension, dyslipidemia, and smoking status. Finally, the Locally Weighted Scatter-Plot Smoother (LOWESS) function was used to graphically depict the predicted probability of a median longer positivity duration and the variables of interest. Statistical analysis was performed using Stata version 16.1 (Stata Corporation, College Station, TX, USA).

## 3. Results

A total number of 25 patients with a median age of 70 years (IQR: 57–78) and a median ACCI value of 3 (IQR: 2–5) were considered eligible for final analysis. The median duration of positive viral RT-PCR for the whole cohort was 20 days (IQR, 16–26). Patients requiring invasive oxygenation support at hospital admission (Ventimask, *n* = 9; CPAP, *n* = 7) were associated with a significantly longer median viral positivity duration (25 days, IQR: 19–35 vs. 12 days, IQR: 10–19, *p* = 0.001) and lower median TT serum levels (2.64 ng/mL, IQR: 1.56–2.99 vs. 5.4 ng/mL, IQR: 4.47–5.56, *p* = 0.011). Patients’ hemato-chemical and clinical characteristics, stratified according to impaired TT levels (i.e., <3 ng/mL) at the moment of hospital admission, are presented in Table 1.

**Figure 1:**
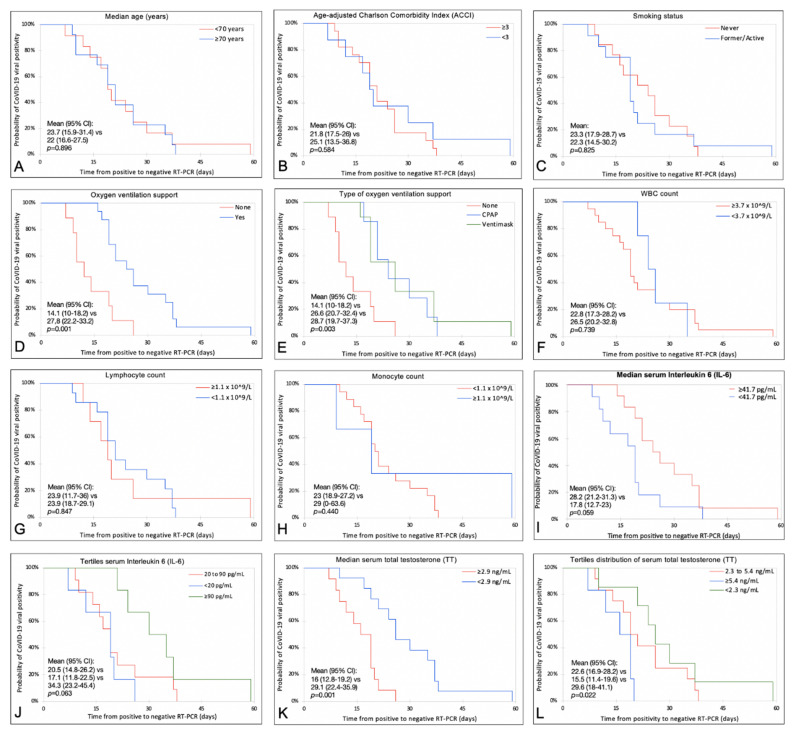
Kaplan–Meier estimates and Log-rank test to explore differences between clinical variables for the duration of COVID-19 viral positivity. Comparisons between: (**A**) = patient age groups; (**B**) = ACCI groups; (**C**) = smoking status; (**D**) = oxygen ventilation support; (**E**) = type of oxygen ventilation; (**F**) = WBC count; (**G**) = lymphocyte count; (**H**) = monocyte count; (**I**) = IL-6; (**J**) = tertiles distribution of IL-6; (**K**) = serum TT; (**L**) = tertiles distribution of TT. ACCI: Age-Adjusted Charlson Comorbidity Index; WBC: white blood cell; IL-6: interleukin 6; TT: total testosterone.

**Figure 2:**
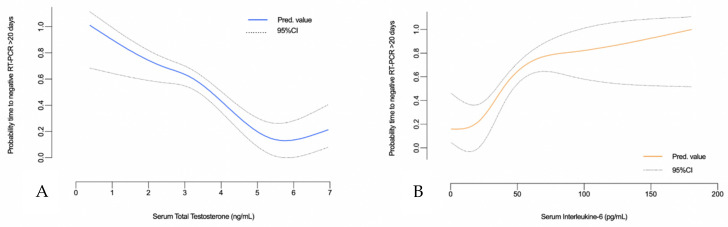
Multivariable adjusted Locally Weighted Scatter-Plot Smoother (LOWESS) function depicting the predicted probability of time to negative RT-PCR > 20 days and the increasing levels of serum total testosterone (**A**) and Interleukine-6 (**B**) detected at hospital admission.

Kaplan–Meier and Log-rank estimates of the peripheral blood cell value, immune cell subset, or available cytokine interleukin, as well as serum TT level vs. duration of viral positivity, are summarized in Figure 1. Of note, both the need for oxygen support and lower serum TT levels were associated with a longer time to negative RT-PCR test (mean positivity duration: 27.8 days, 95% CI: 22.2–33.2 vs. 14.1 days, 95%CI: 10–18.2, *p* = 0.001; 29.2 days, 95%CI: 22.5–35.9 vs. 16 days, 95%CI: 12.9–19.2, *p* = 0.001 respectively; Figure 1D,K). Similarly, the univariate Cox regression model confirmed, among all the covariates tested, that the need for O_2_ support, highest IL-6 values (i.e., ≥90 pg/mL) as well as increasing TT levels were associated with a significantly shorter viral positivity duration (HR_O2_: 0.23, 95%CI: 0.19–0.59, HR_IL-6_, 0.25, 0.11–0.90 and HR_TT_ 1.47 95%CI: 1.11–1.94, Table 2). On multivariable analysis, both higher baseline TT levels and the need for oxygen supplementation were found independently associated with shorter and longer viral positivity durations (HR_TT_: 1.39, 95%CI: 0.95–2.03 and HR_O2_: 0.19, 95%CI: 0.03–1.18 respectively; Table 2). Furthermore, increasing TT levels were found to be associated with a shorter duration of viral positivity even when adjusted for specific clinic-demographic confounders such as ACCI, history of hypertension, dyslipidemia, and smoking status (aHR: 1.34, 95%CI: 1.02–1.88, *p* = 0.035). The probability of having a longer duration (i.e., greater than the median of 20 days) showed a linear and inverse relationship with the increasing values of serum TT and IL-6 (Figure 2).

## 4. Discussion

To date, only a few studies have focused on the duration of viral positivity of SARS-CoV-2 during infection [15]. From an epidemiological standpoint, the duration of viral positivity is a key factor for the control of the pandemic and for making decisions regarding resource utilization and isolation precautions. Viral positivity has been described to persist for an interval varying between 10 and 60 days after symptoms’ onset [16,17]. The large variability in the duration of viral positivity leads to the speculation of risk factors for variability in the clinical presentations of the disease (e.g., asymptomatic/paucisymptomatic vs. moderate or severe ill conditions) and therefore to the immune response to infection.

In the current study, the median viral positivity duration for the whole cohort was 20 days (IQR, 16–26). This duration is longer than some previous studies on this topic. For example, Young BE et al. observed a median duration of viral positivity of 12 days (range, 1–24 days) in a population of 18 patients with COVID-19 infection [18]. Similarly, Lin A et al. reported a median SARS-CoV-2 viral positivity duration of 12 days (range 4–45 days) in a cohort of 137 patients with predominantly mild to moderate disease [19]. Given the potential differences among these studies in the time of performing RT-PCR during the follow-up, the longer duration of viral positivity in our study could partly be explained by some considerations. First, the median age of the current patient population is significantly older (70 years, QR: 57–78) than in the studies by Young BE et al. [18] (47 years, range 31–73) and Lin A et al. (47 years, range 4–83) but comparable to other clinical experiences of hospitalized patients in Italy [20]. In this context, age has been reported as a significant risk factor for the longer duration of viral positivity in several experiences with a higher risk for patients [21,22]. Moreover, the severity of the disease has also been reported to be associated with a long duration of viral positivity [19,21,23]. Given that an association between severe disease and a long time of viral positivity duration can be intuitive, in our analysis, more severe SARS-CoV-2 presentation (i.e., the need for invasive oxygen assistance) was associated with a significantly longer viral positivity duration. Considering the small population of our analysis (*n* = 25), which represents the major limitation of our study, we believe that these data suggest that more complex interactions exist for viral positivity duration including the immune system and hormonal constitution.

In this study, we were able to evaluate the association of testosterone levels with the duration of viral positivity. Interestingly, in our analysis, increasing serum TT levels (ng/mL) were independently associated with shorter viral positivity duration (HR: 1.34, 95%CI: 1.02–1.88). Recently, testosterone has received great attention even in the context of the COVID-19 pandemic for its effects on the general health status and for modulating the transmembrane serine protease 2 (TMPRSS2), which plays a crucial role in the entry of the SARS-CoV-2 virus into the respiratory epithelial cells leading to the COVID-19 disease [24,25]. TMPRSS2 is expressed in prostate epithelium and is regulated by the androgen receptor (AR) [26]. All these functions have led investigators to hypothesize a key role of testosterone and, consequently, of androgen deprivation therapy (ADT), in patients with COVID-19 infection [27,28]. Despite the modulating action of testosterone on TMPRSS2, which has led to the hypothesis there being a role of ADT in patients with COVID-19, the currently available evidence has shown conflicting results [29,30]. Previous experiences showed that low testosterone levels were independently associated with worse overall men’s health and clinical COVID-19 phenotype, need for intensive care unit (ICU) treatment, and mortality [10,11,30,31,32,33,34,35]. Moreover, an inverse correlation was found between testosterone levels and IL-6 levels [11], suggesting that testosterone could play an important role in both early and late stages of disease [8]. On the basis of these results, it has been hypothesized that testosterone may play a double-edged role in the pathogenesis of the COVID-19 disease. In the early phase, the immunosuppressive action of testosterone could explain males’ greater susceptibility to infection when compared to women in all age groups. When the infection has settled, in elderly males who frequently develop a severe course, lower testosterone levels related to age could result in a lower immunosuppressive effect on the cytokine storm [8]. Given the prior evidence on the role of testosterone in the clinical course of the COVID-19 disease, in this study, we hypothesized and confirmed that testosterone could also be important in the duration of viral positivity. Contrary to our findings, Xu H et al. did not observe a significant correlation between TT and duration of viral positivity in a cohort of 39 patients with mild to critical disease [36]. However, some differences between the two studies should be highlighted. First, in the study by Xu H et al., long-term positivity was defined as the duration of viral positivity >50 days comparing with our study in which a rigid definition of long viral positivity was not given. Second, TT levels and other sex-related hormones like FSH and LH were in the normal reference ranges in all patients with the COVID-19 disease (median TT levels 3.93 ng/mL) and in the control group (median TT levels 3.83 ng/mL) in the Xu study, which did not allow the definition the impact of very low testosterone values, like hypogonadism status, on the disease duration. In contrast, in our study, median TT serum levels were 2.64 ng/mL, IQR: 1.56–2.99 in the group with longer times till negative RT-PCR tests vs. 5.4 ng/mL, IQR: 4.47–5.56, in the group with shorter times till negative RT-PCR *p* = 0.011). To our knowledge, our study was the first to demonstrate an association between TT levels and the duration of viral positivity.

We believe that the results of our study should lead to better defining the role of testosterone levels in the pathogenesis of the COVID-19 disease. It is known that hypogonadism is associated with male health status and recently great attention has been given to its role in the context of COVID-19, given the sex differences in disease outcomes [37,38,39]. It is important to note that this study has also several limitations. First, the retrospective design and limited sample size expose the current analysis to bias and the role of chance. Second, our data allowed us only to make implications on the duration of viral positivity at hospital admission but not to define the role of TT in the pathogenesis of the disease. Third, we did an analysis only of hospitalized patients with mild to severe disease and not on asymptomatic patients. Additionally, we were able to retrospectively collect data only from a single blood draw at the moment of hospital admission for serum total testosterone without the possibility of exploring the biochemical and clinical implications on the fraction of free and bioavailable proportion or a confirmatory second sample. This particular aspect will require further evaluation in future prospective and possibly multicentered experiences. Finally, our hypothesis has been developed with regard to the male sex given androgens play a crucial role in the lifespan of a male subject. Nevertheless, the role of testosterone could potentially provide new insights into the progression of SARS-CoV-2 disease in female patients. We believe that these data should be analyzed in the future as they can be crucial for implementing pandemic control strategies.

## 5. Conclusions

Our findings suggest that testosterone values may be associated with the duration of viral positivity and thus could be used to predict viral clearance to help pandemic control strategies. In particular, baseline higher total testosterone levels for male COVID-19 patients at hospital admission are associated with shorter durations of positive COVID-19 testing and thus more rapid viral clearance. Importantly, further studies are needed to understand the role of testosterone in the pathogenesis of the disease.

## Figures and Tables

**Table 1:** Clinical and hemato-chemical characteristics of COVID-19 patients at hospital admission according to baseline total testosterone levels.

Variables	Median Total Testosterone at Hospital Admission		*p*-Value *
	<2.9 ng/mL	≥2.9 ng/mL	
Sample size, n (%)	13 (52)	12 (48)	
Age, years	70 (66–78)	70 (41–77)	0.68
ACCI score, median (IQR)	3 (2–5)	3 (1–5)	0.93
Time to negative RT-PCR, days, median (IQR)	26 (21–37)	18 (12–19)	**0.002**
Requiring O_2_ assistance:			**0.007**
None	1 (8.3)	8 (61.5)	
Ventimask	6 (50)	3 (23.1)	
CPAP	6 (50)	1 (7.7)	
**Prevalence of comorbidities**, *n* (%)			
Hypertension	7 (53.8)	6 (50)	0.85
Diabetes	3 (23.1)	4 (33.3)	0.57
Dyslipidemia	1 (7.7)	4 (33.3)	0.11
History of Neoplasm	0	3 (25)	0.06
CVD	2 (15.4)	2 (16.7)	0.93
CKD	2 (15.4)	0	0.16
Lung disease	3 (23.1)	1 (8.3)	0.36
Smoking status	5 (38.5)	7 (58.3)	0.32
**Complete Blood Count**, median (IQR)			
HGB, g/dL Nr: 12–15.5	12.7 (11.0–14.5)	13.5 (12.5–15.0)	0.09
HCT, % Nr: 41–50	45.7 (40.0–47.0)	46.5 (41.0–48.0)	0.67
WBC, ×10^9^/L Nr: 3.70–11.30	7.3 (3.6–13.6)	7 (5.3–7.9)	0.82
PLT, ×10^9^/L Nr: 150.0–450.0	208.5 (180.5–268.5)	200.0 (183.0–240.0)	0.78
Lymphocytes, ×10^9^/L Nr: 1.10–4.80	1.1 (0.7–1.2)	0.7 (0.5–1)	0.32
Lymphocytes CD4+, *n*°/μL Nr: 410.0–1590.0	620.0 (375.5–773.5)	524.5 (282.8–629)	0.54
Lymphocytes CD4+, % Nr: 31.0–60.0	45.3 (41.3–51.8)	36.0 (34.4–45.9)	0.40
Lymphocytes CD8+, *n*°/μL Nr: 190.0–1140.0	330.0 (277.5–507)	237.0 (137–550.3)	0.39
Lymphocytes CD8+, % Nr: 13.0–41.0	27.7 (19.8–36.5)	23.3 (17.7–32.5)	0.46
CD4+/CD8+, ratio Nr: 0.60–2.80	1.1 (1.0–2.6)	1.1 (1.1–2.7)	0.89
NK cells, *n*°/μL Nr: 150.0–590.0	154.0 (137.5–172.0)	59.0 (44.0–190.0)	0.22
NK cells, % Nr: 5.0–27.0	13.8 (9.8–15)	6.5 (4.0–10.9)	0.18
Lymphocytes B, *n*°/μL Nr: 90.0–660.0	187.0 (126.0–235.0)	113.0 (95.0–115.0)	0.17
Lymphocytes B, % Nr: 6.0–25.0	13.1 (9.0–14.1)	8.2 (6.0–11.1)	0.23
Monocytes, ×10^9^/L Nr: 0.10–1.10	0.2 (0.2–0.5)	0.4 (0.4–0.7)	**0.025**
Monocytes, % Nr: 3.5–10.5	8.0 (7.1–8.9)	3.4 (2.9–5.8)	**0.012**
**Blood chemistry**, median (IQR)			
Creatinine, mg/dL Nr: 0.70–1.20	0.9 (0.7–1.1)	1.0 (0.8–1.2)	0.477
Testosterone, ng/mL Nr: 2.80–8.00	2.3 (1.4–2.7)	5.5 (4.4–5.9)	**<0.0001**
IL-6, pg/mL Nr: 1.50–7.00	91.2 (40.2–99.0)	27.2 (5.4–44.9)	**0.006**
CRP, mg/dL Nr: 0.00–0.50	14.4 (6.5–21.0)	3.3 (1.1–4.7)	**0.003**
LDH, U/L Nr: 135.0–225.0	357 (268–493)	224 (210–334)	0.186
Lac, mmol/L Nr: 0.3–0.7	1.2 (1.0–1.5)	0.9 (0.7–1.3)	0.286
Na^+^, mmol/L Nr: 136.0–145.0	135.0 (132.0–137.0)	138.0 (133.5–140.0)	0.077
K^+^, mmol/L Nr: 3.40–5.50	3.9 (3.6–4.3)	3.8 (3.5–4.3)	0.941
D-Dimer, ng/mL Nr: <500	1285 (957.8–2906)	840 (402.3–1304.5)	0.082
**Vital signs**, median (IQR)			
pH Nr: 7.35–7.45	7.49 (7.48–7.50)	7.44 (7.42–7.48)	0.119
pO_2_, mmHg Nr: 83.0–108.0	73 (60.0–86.0)	101.0 (77.0–113.0)	0.092
PaO_2_/FiO_2_, mmHg Nr: 200–400	288.0 (272.0–359.0)	480.0 (364.0–491.0)	0.065
SO_2_, % Nr: 94.0–98.0	97.0 (95.0–99.0)	97.0 (95.0–98.0)	0.879

Results are presented as *n* (%) or median (range). ACCI Age-Adjusted Charlson Comorbidity Index, RT-PCR real-time reverse transcriptase–polymerase chain reaction, CPAP continuous positive airway pressure, CVD cardiovascular disease, CKD chronic kidney disease, WBC white blood cells, PLT platelets, NK natural killer, IL-6 interleukin-6, CRP C-reactive protein, LDH lactate dehydrogenase, Lac lactate. * *p*-values according to Fisher’s Exact test or Mann–Whitney U test when appropriate.

**Table 2:** Cox Proportional Hazards Model assessing variables influencing time till negative RT-PCR.

Subgroups and/or Continuous Variables		Univariate Analysis		Multivariate Analysis *	
		HR (95%CI)	*p*-Value	HR (95%CI)	*p*-Value
Age, years	<70	Ref	--		
	≥70	1.05 (0.47–2.35)	0.9		
ACCI	<3	Ref	--		
	≥3	1.27 (0.52–3.09)	0.61		
Requiring O_2_ support at admission	No	Ref	--	Ref	--
	Yes	0.23 (0.16–0.59)	**0.002**	0.19 (0.03–1.18)	**0.08**
O_2_ ventilation strategy	None	Ref	--	Ref	--
	Ventimask	0.21 (0.13–0.62)	**0.004**	0.36 (0.10–1.38)	0.14
	CPAP	0.25 (0.18–0.74)	**0.012**	0.42 (0.11–1.69)	0.22
WBC, ×10^9^/L	<3.7	Ref	--		
	≥3.7	1.20 (0.40–3.60)	0.75		
WBC, ×10^9^/L	Continuous	0.94 (0.87–1.03)	0.18		
Lymphocyte, ×10^9^/L	<1.10	Ref	--		
	≥1.10	1.10 (0.42–2.89)	0.85		
Lymphocyte, ×10^9^/L	Continuous	0.83 (0.53–1.30)	0.42		
Lymphocytes CD4+, *n*°/μL	<410	Ref	--		
	≥410	0.85 (0.28–2.55)	0.77		
Lymphocytes CD4+, *n*°/μL	Continuous	0.99 (0.98–1.11)	0.43		
Lymphocytes CD8+, *n*°/μL	<190.0	Ref	--		
	≥190.0	1.45 (0.49–4.26)	0.50		
Lymphocytes CD8+, *n*°/μL	Continuous	1.0 (0.99–1.10)	0.99		
Monocyte, *x*10^9^/L	<1.10	Ref	--		
	≥1.10	0.57 (0.13–2.57)	0.47		
Monocyte, *x*10^9^/L	Continuous	1.09 (0.93–1.27)	0.27		
Lymphocytes B, *n°/*μL	<90.0	Ref	--		
	≥90.0	0.99 (0.25–3.91)	0.99		
Lymphocytes B, *n°/*μL	Continuous	1.0 (0.99–1.0)	0.29		
CD56+ NK, *n°/*μL	<100.0	Ref	--		
	≥100	1.33 (0.41–4.27)	0.64		
CD56+ NK, *n°/*μL	Continuous	1.0 (0.99–1.0)	0.32		
Median serum IL-6, pg/mL	<42	Ref	--		
	≥42	0.46 (0.19–1.09)	0.08		
Tertiles serum IL-6, pg/mL	<20	Ref	--	Ref	--
	20–90	0.61 (0.21–1.76)	0.36	4.47 (0.69–29.08)	0.12
	≥90	0.25 (0.11–0.90)	**0.034**	2.42 (0.28–21.36)	0.43
Serum IL-6, pg/mL	Continuous				
Median serum TT, ng/mL	<2.9	Ref	--	Ref	--
	≥2.9	0.21 (0.10–0.58)	**0.002**	0.34 (0.10–1.33)	0.12
Tertiles serum TT, ng/mL	<2.3	Ref	--	Ref	--
	2.3–5.4	1.65 (0.61–4.40)	0.32	1.41 (0.40–4.97)	0.60
	≥5.4	5.14 (1.40–18.93)	**0.014**	5.14 (0.88–30.12)	**0.07**
Serum TT, ng/mL	Continuous	1.47 (1.11–1.94)	**0.007**	1.39 (0.95–2.03)	**0.09**

ACCI Age-Adjusted Charlson Comorbidity Index, RT-PCR real-time reverse transcriptase–polymerase chain reaction, CPAP continuous positive airway pressure, CVD cardiovascular disease, CKD chronic kidney disease, WBC white blood cells, PLT platelets, NK natural killer, IL-6 interleukin-6. * Entering and removing limit set at *p* = 0.05 and *p* = 0.10 respectively.
